# P-1018. Diagnostic Ambiguity in Clostridioides difficile Testing and a Call for Standardization

**DOI:** 10.1093/ofid/ofaf695.1214

**Published:** 2026-01-11

**Authors:** Perani V Chander, Jennifer L Johnson, Jennifer M McDaniel, Erin Moyer, George Youssef, Suganthini Krishnan Natesan, Ryan Kuhn, Sorabh Dhar

**Affiliations:** Wayne State University School of Medicine/Detroit Medical Center, Detroit, MI; John D. Dingell, VAMC, Brownstown, MI; John D Dingell VAMC, Detroit, Michigan; John D Dingell VAMC, Detroit, Michigan; John D Dingell VAMC, Detroit, Michigan; John D. Dingell VA Medical Center, Wayne State University, OAKLAND TOWNSHIP, Michigan; John D Dingell VA Medical Center, Detroit, Michigan; Wayne State University/Detroit Medical Center, John Dingell VAMC, Detroit, MI

## Abstract

**Background:**

The Centers for Disease Control and Prevention (CDC) outlines 4 testing options for the diagnosis of *Clostridioides difficile* infections (CDI), including nucleic acid amplification tests (NAAT), glutamine dehydrogenase antigen (GDH Ag), *C. difficile* toxin, and stool cell culture cytotoxin assays. Cytotoxin assay is considered the gold standard test but has limited use in real time diagnosis. Healthcare centers report CDI to the National Healthcare Safety Network (NHSN), per the LabID reporting guidelines. This often leads to significant variability in reporting rates and clinical diagnosis. The CDC has proposed a new algorithm accounting for clinical judgement in the form of antibiotics for CDI treatment for at least 5 days and appropriate testing.

**Figure:**
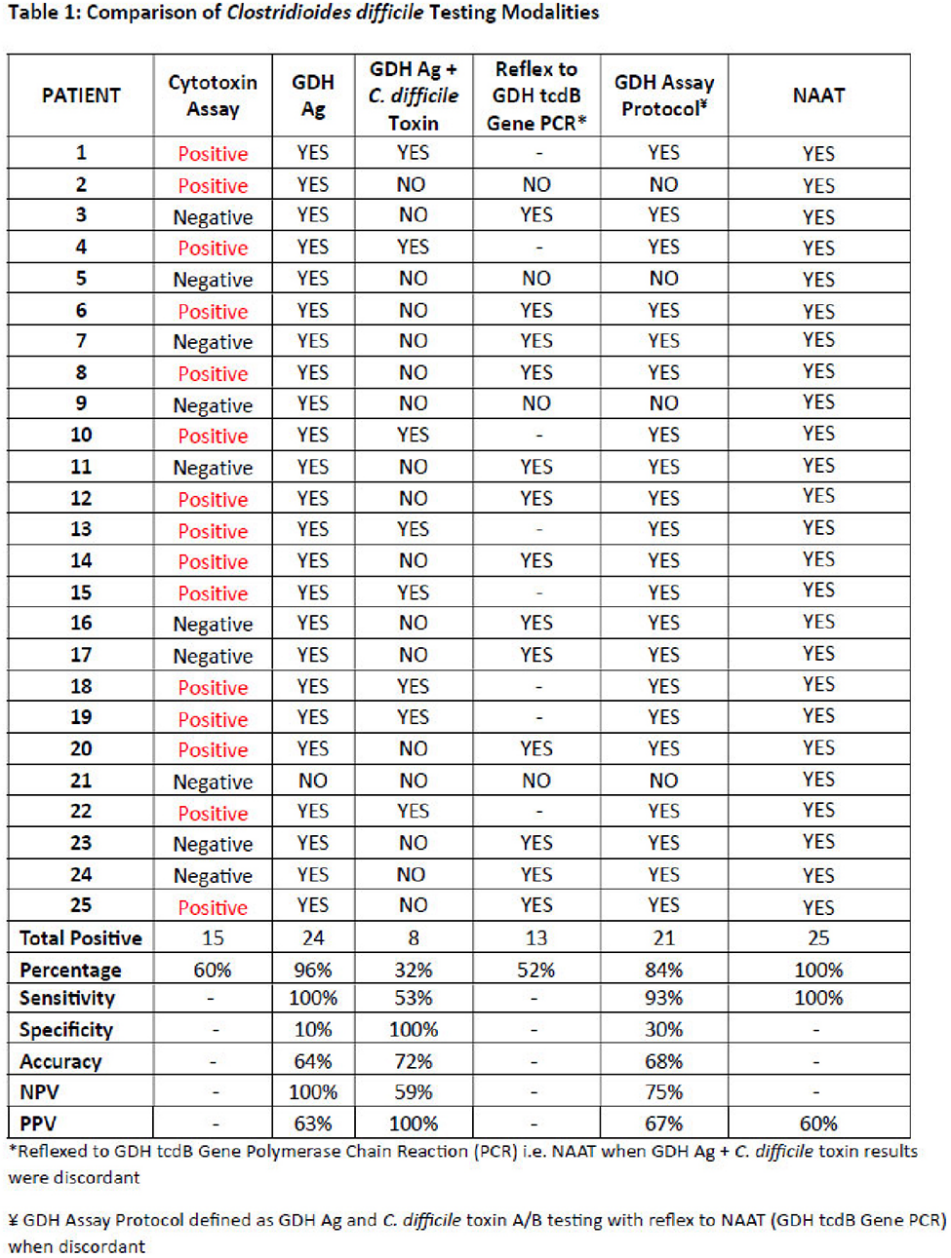

**Figure:**
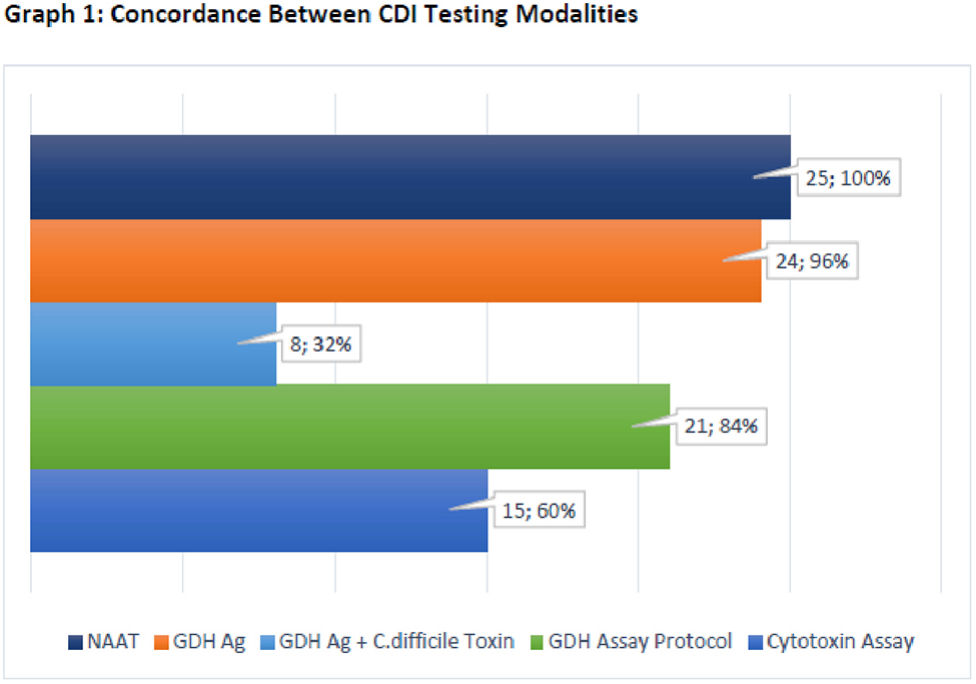

**Methods:**

This single center, quasi-experimental study compares the different testing modalities, with clinical correlation for CDI. Patient samples that tested positive for routine NAAT from 9/30/2023 to 4/5/2025 were concurrently sent for cytotoxin assay, GDH Ag and *C. difficile* toxin A/B testing with reflex to NAAT (the GDH assay protocol). Clinical charts were reviewed for risk factors, clinical presentation, antimicrobial utilization, and outcomes. Statistical analysis was performed using the cytotoxin assay as the gold standard.

**Figure:**
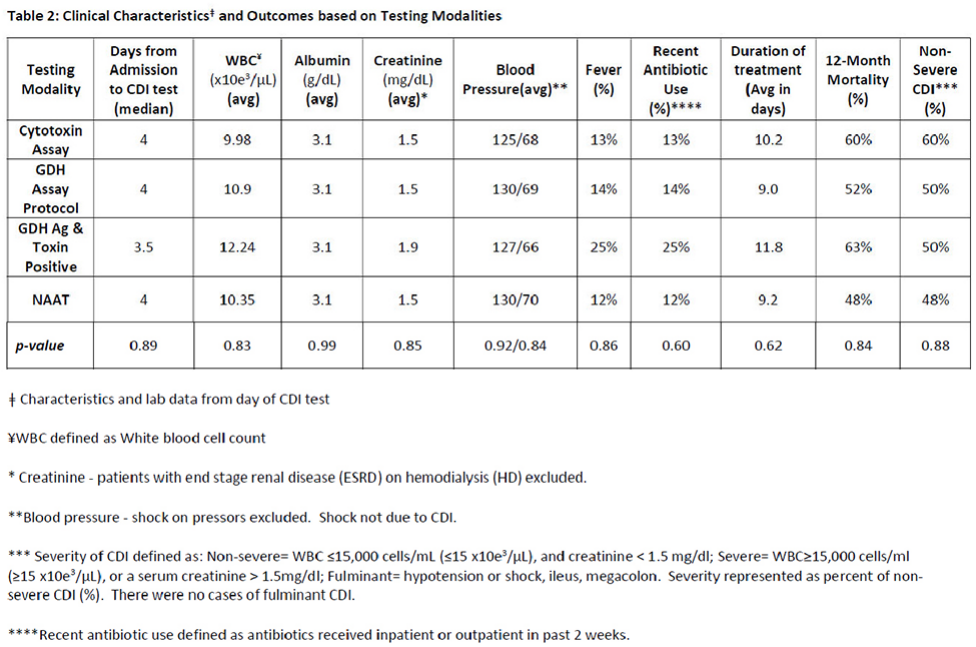

**Results:**

Of the 25 specimens that tested positive for CDI by NAAT, 15 (60%) were positive on the cytotoxin assay, 24 (96%) were positive for GDH Ag, 8 (32%) were positive for *C. difficile* toxin and GDH Ag, 21 (84%) were positive using the multi-step GDH assay protocol (table 1, graph 1). Clinical parameters and outcomes are notable for a median age of 72, a median of 4 (IQR 5) days to testing from admission, most commonly presenting as non-severe CDI in 48%, recent antibiotic use in 64%, and a mortality of 48% (table 2). The addition of qualifying antibiotics for a duration of at least 5 days to the CDI definition resulted in a positive result of 84% for samples tested by the GDH and NAAT only assays.

**Conclusion:**

A high degree of variability was noted when comparing different testing modalities that could lead to inaccurate diagnostic results and confound national reporting of CDI rates. A need for standardization of CDI testing to include additional factors (i.e. antibiotic duration, qualified tests) is urgently needed.

**Disclosures:**

All Authors: No reported disclosures

